# Exosomes: an innovative therapeutic target for cerebral ischemia-reperfusion injury

**DOI:** 10.3389/fphar.2025.1552500

**Published:** 2025-03-26

**Authors:** Yuan Yang, Yushan Duan, Jinxi Yue, Yue Yin, Yiming Ma, Xiaohong Wan, Jianlin Shao

**Affiliations:** ^1^ Department of Critical Care Medicine, The Second Affiliated Hospital, Kunming Medical University, Kunming, China; ^2^ Department of Anesthesiology, The First Affiliated Hospital, Kunming Medical University, Kunming, China

**Keywords:** cerebral ischemia-reperfusion injury, exosomes, mesenchymal stem cells, mitochondria, autophagy

## Abstract

Ischemic stroke is caused by artery stenosis or occlusion, which reduces blood flow and may cause brain damage. Treatment includes restoring blood supply; however, ischemia-reperfusion can still aggravate tissue injury. Reperfusion injury can increase levels of reactive oxygen species, exacerbate mitochondrial dysfunction, create excessive autophagy and ferroptosis, and cause inflammation during microglial infiltration. Cerebral ischemia-reperfusion injury (CIRI) is a key challenge in the treatment of ischemic stroke. Currently, thrombolysis (e.g., rt-PA therapy) and mechanical thrombectomy are the primary treatments, but their application is restricted by narrow therapeutic windows (<4.5 h) and risks of hemorrhagic complications. Exosomes reduce CIRI by regulating oxidative stress, mitochondrial autophagy, inflammatory responses, and glial cell polarization. In addition, their noncellular characteristics provide a safer alternative to stem cell therapy. This article reviews the research progress of exosomes in CIRI in recent years.

## 1 Introduction

### 1.1 Cerebral ischemia-reperfusion injury

Stroke is an abrupt cerebrovascular incident resulting from interruptions in the local blood circulation to the brain, and it stands as the second most prevalent cause of mortality worldwide (Li et al., 2024). According to a 2017 World Health Organization report, approximately 6.24 million individuals die from stroke annually. In China, it is projected that there exists around 2.4 million newly diagnosed stroke cases and approximately 1.1 million fatalities associated with strokes each year, with 75%–85% attributed to ischemic strokes (Wang et al., 2022). Ischemic stroke arises from the narrowing or the blockage of cerebral vascular supply arteries, causing insufficient blood supply to the brain, leading to local brain tissue disintegration and damage (Feigin et al., 2025). It is a prevalent acute cerebrovascular condition and the principal reason for death among middle-aged and elderly populations (Ly and Maquet, 2014). Cerebral ischemia refers to a disruption in the circulation of blood to the brain, triggering intricate metabolic and cellular pathologies that lead to neuronal cell death and cerebral infarction (Shin et al., 2020). The intensity of localized brain injury resulting from cerebral ischemia is contingent upon the length of the ischemic episode. Although transient symptoms of cerebral ischemia may suggest reversible impairment, prolonged ischemic and hypoxic conditions may result in the development of cerebral edema and subsequent neuronal cell death (HU et al., 2016). Standard therapeutic approaches for ischemic cerebrovascular disorders encompass mechanical thrombectomy along with the intravenous delivery of tissue plasminogen activator. These interventions aim to swiftly restore blood circulation to the affected ischemic brain regions (Stegner et al., 2019; Wu et al., 2020). Nevertheless, reinstating blood circulation to the ischemic area, referred to as ischemia-reperfusion injury (IRI), may also result in damage to brain tissue. Reperfusion injury is cellular harm occurring upon the restoration of blood supply following ischemia or hypoxia, potentially causing swelling and oxidative injury rather than a return to normal function (Li et al., 2017). Clinical investigations have associated the advancement of CIRI with free radicals, intracellular calcium excess, leukocyte attachment and clustering, and inadequate production of high-energy phosphate molecules (Liu R. et al., 2017). Therefore, developing effective treatments to mitigate brain damage resulting from CIRI is urgently required.

### 1.2 Introduction to exosomes

**FIGURE 1:**
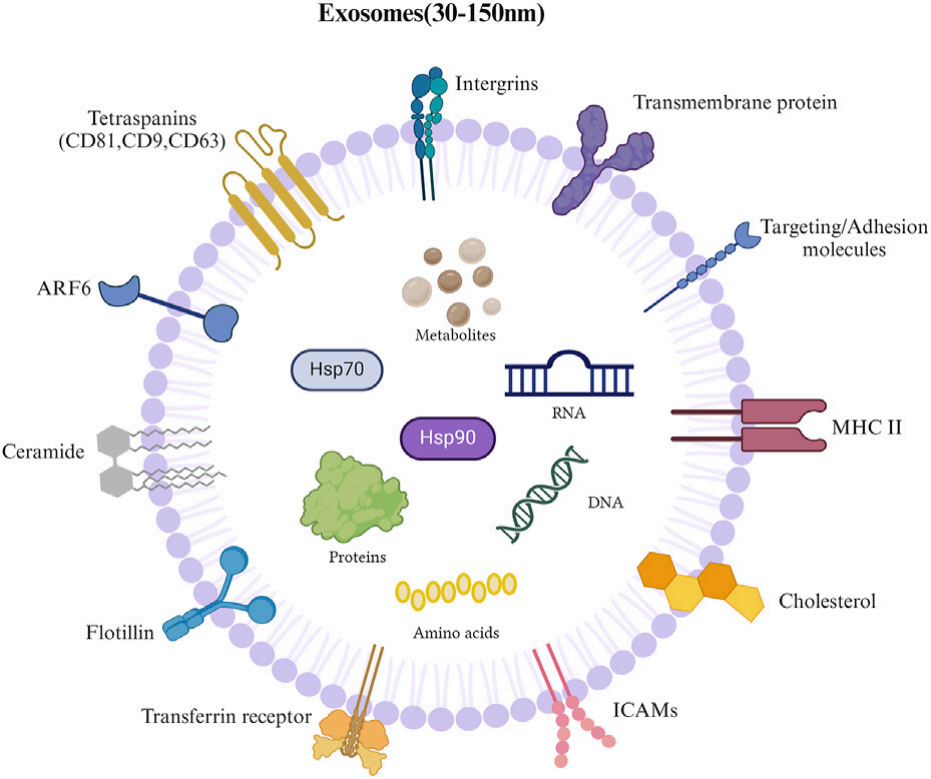
Composition of exosomes. 1) Lipids: lipid bilayer that forms exosomal membrane; 2) Nucleic acids: DNA and RNA; 3) Immune regulatory molecules: major histocompatibility complex; 4) Proteins: transmembrane proteins (CD9, CD63, CD81), intercellular adhesion molecules, integrins, and transferrin receptors; 5) Metabolites.

Exosomes are extracellular vesicles measuring 30–150 nm in diameter, secreted by different cell types and commonly present in biological fluids (Van Niel et al., 2018). They contain various biologically active substances, such as proteins, lipids, DNA, and RNA (Pefanis et al., 2015; Van Niel et al., 2018). Exosomes can permeate to adjacent cells or be conveyed to remote anatomical sites, where they transmit signals or information to particular recipient cells (Li L. et al., 2016; Hsu et al., 2017). Exosomes, derived from late endosomal compartments, are nanoscale, disc-shaped structures with low immunogenicity and high compatibility (Guo et al., 2021). They correspond to vesicles within the lumen of multivesicular bodies, which are discharged into the environment as exosomes when multivesicular bodies merge with the plasma membrane. Extracellular vesicles, rich in exosomes, contain coding and non-coding RNA, lipids, and proteins and play an essential role in intercellular communication (Witwer et al., 2013; Théry et al., 2006; Valadi et al., 2007; Ludwig and Giebel, 2012). Exosomes and other extracellular vesicles have several common characteristics, such as their remarkable stability in circulation, detectability in intricate biological fluids, and molecular composition functioning as a “liquid biopsy.” These qualities make them excellent biomarkers for diseases (Bautista-López et al., 2017; Nedaeinia et al., 2017; Théry, 2015). As cell-released carriers, exosomes play a crucial role in communication between various cell types (Yang et al., 2019). The surface of extracellular vesicles is rich in specific protein markers (such as CD9, CD63, CD81, and TSG101), which participate in exosome biogenesis and secretion regulation. For example, CD47 evades immune clearance through a “don’t eat me” signal, which enhances the stability of extracellular vesicles *in vivo*; HSP70 and HSP90 help exosomes adapt to the extracellular environment; and MHC molecules mediate antigen information transmission between immune cells. In addition, exosomes carry donor cell-specific proteins (such as synaptophysin, which is expressed in neuronal derived exosomes), and thus provide a molecular basis for targeted therapy (Kalluri and LeBleu, 2020; Welsh et al., 2024; Luan et al., 2025) (Figure 1).

### 1.3 Therapeutic prospects of exosomes

Currently, efficient therapeutic alternatives for ischemic stroke are limited and are exclusively appropriate for a limited number of stroke patients. Urokinases and streptokinases are potential neuroprotective agents that have been clinically utilized for the management of ischemic stroke. While these medications can activate plasminogen to generate plasmin, they also have the capacity to degrade coagulation factors and fibrinogen, heightening the risk of hemorrhage when administered as thrombolytic therapy (Hacke et al., 2008). The U.S. Food and Drug Administration (FDA) has demonstrated that a genetically engineered tissue plasminogen activator can eliminate obstructed blood vessels to address ischemic stroke; nevertheless, due to the limited treatment window (<4.5 h), its therapeutic effect is limited, and this treatment option increases the risk of intracranial hemorrhage (Broeg-Morvay et al., 2016). Treatment strategies targeting inflammation from CIRI have been successful in animal models but have not yet applied in clinical practice (Liu et al., 2019; Howell et al., 2023). The exceedingly stringent blood-brain barrier (BBB) and the “no-reflow” phenomenon following CIRI complicate the entry of pharmaceuticals into the brain parenchyma. Among them, the no-reflow phenomenon refers to the obstruction of microcirculation perfusion after vascular recanalization. To tackle this challenge, nanotechnology-driven drug delivery systems have been engineered and have demonstrated encouraging outcomes (Gabathuler, 2010; Chen et al., 2020). Nevertheless, administering adequate therapeutic agents to the brain continues to pose a considerable challenge for nanomaterials. Kozlovskaya et al. illustrated that the majority of nanocarrier systems can transport fewer than 1% (median) of the injected dosage to the brain (Kozlovskaya and Stepensky, 2013). Additionally, achieving brain penetration and prolonging retention time often require costly and labor-intensive modifications with peptides or antibodies for clinical applications. Therefore, developing alternative therapies for acute ischemic stroke patients is urgently needed (Li et al., 2020).

CIRI injury is a complex pathophysiological mechanism characterized by energy disruption, cellular acidosis, increased excitatory amino acid release, disturbed intracellular calcium equilibrium, reactive oxygen species generation, and initiation of apoptotic gene expression. These factors are interconnected, establishing a detrimental cycle that ultimately results in cellular apoptosis or necrosis (Hankey, 2019). Prior research has demonstrated that human mesenchymal stem cells (MSCs), human bone marrow-derived MSCs, human umbilical cord blood-derived MSCs, human adipose tissue-derived MSCs, and human neural stem cells possess significant therapeutic potential in the treatment of stroke (Chen et al., 2001; Nomura et al., 2005; Song et al., 2017; Wang et al., 2017; Boese et al., 2018). Stem cell therapy for the management of ischemic stroke can enhance endogenous neural regeneration and brain tissue self-repair. However, the therapeutic efficacy of stem cells is affected by their heterogeneity. Additionally, certain stem cell therapies present safety hazards, chiefly encompassing tumorigenicity, atypical immune reactions, and unintended differentiation (Zheng et al., 2018). Exosomes are increasingly recognized for their role as transporters of functional proteins, lipids, and nucleic acids (Li et al., 2023). Numerous studies have confirmed that exosomes can alleviate CIRI in various ways, suggesting broad application prospects. This review discusses recent advancements in exosome research related to CIRI.

## 2 Mechanism of cerebral ischemia-reperfusion injury

### 2.1 Cerebral ischemia-reperfusion injury and reactive oxygen species

Reactive oxygen species (ROS) are connected to brain injury post-ischemic stroke (Jiang et al., 2020). Marked oxidative stress and inflammation occur after the onset of I/R, producing excessive ROS in brain tissue. This surge in ROS causes oxidative damage to neurons, worsening brain injury, and cerebral infarction (Prakash and Carmichael, 2015; Yang Y. et al., 2015). Evidence also suggests that ROS production in mitochondria rapidly increases post-acute ischemic stroke, leading to impaired mitochondrial function through the decrease of membrane potential and the disruption of mitochondrial membrane integrity (Wang et al., 2019). Subsequently, apoptosis that is dependent on caspase and mediated by mitochondria is initiated in neuronal cells (George and Steinberg, 2015). The continuous accumulation of ROS damages the BBB integrity by promoting the degradation of tight junction proteins, further exacerbating brain injury (Prakash and Carmichael, 2015; Yang Y. et al., 2015). Therefore, research on antioxidant strategies that help reduce oxidative damage is crucial.

### 2.2 Cerebral ischemia-reperfusion injury and mitochondrial dysfunction

Mitochondria play a vital role in preserving cellular dynamic equilibrium by engaging in ongoing processes such as fission, fusion, autophagy, and biogenesis, all of which are essential for the proper functioning of mitochondria (Song et al., 2021). In recent times, the mechanisms of mitochondrial quality control, specifically the processes of fission, fusion, and autophagy, have garnered significant interest in the context of addressing diseases associated with acute ischemic-hypoxic injuries (Ni et al., 2015). Under normal physiological circumstances, the equilibrium between mitochondrial fission and fusion is essential for preserving the typical structure of mitochondria. An overabundance of fission coupled with a decline in fusion processes can result in mitochondrial fragmentation, which in turn may disrupt vital biological functions, including the stability of mitochondrial DNA, energy production, cellular senescence, and apoptosis. Mitochondria function as the central energy hub for the electron transport chain, consuming oxygen to generate ATP, which provides essential energy for cellular and tissue life activities (Raefsky and Mattson, 2017). Mitochondria are essential contributors to the pathophysiological mechanisms underlying acute ischemic stroke. During ischemic injury, mitochondria serve an essential function in regulating cellular apoptosis through the modulation of reactive oxygen species (ROS) production and calcium levels, as well as by overseeing inflammatory responses and the activation of the inflammasome (Lin and Beal, 2006; Nunnari and Suomalainen, 2012). Excessive mitochondrial damage during acute ischemic stroke can lead to insufficient ATP supply, release of pro-apoptotic factors, and excessive calcium, ultimately causing neuronal death and impacting neuronal function (Andrabi et al., 2020). Haileselassie et al. demonstrated that excessive mitochondrial fission may increase vascular permeability, disrupt the BBB, and induce infectious encephalopathy (Haileselassie et al., 2020). Studies have shown that mitophagy and excessive mitochondrial fission can lead to mitochondrial dysfunction, reducing ATP synthesis, increasing ROS levels, and decreasing mitochondrial membrane potential, ultimately causing cardiovascular and intestinal barrier dysfunction (Duan et al., 2019; 2020b; 2020a). Acute ischemic injury affects mitochondrial quality balance, resulting in excessive mitochondrial fission and abnormal mitophagy (Anzell et al., 2018). Mitochondrial dysfunction is closely associated with CIRI and serves as a significant objective for neuronal cell death following ischemia. Currently, the mechanisms that initiate the harmful cycle of CIRI are still under investigation (Nakamura et al., 2020). Investigating the mechanisms by which mitochondrial damage triggers CIRI injury is crucial for interrupting this cycle and developing effective interventions.

### 2.3 Cerebral ischemia-reperfusion injury and autophagy

Autophagy is a conserved intracellular catabolic route that delivers substantial quantities of cytoplasm, impaired organelles, enduring proteins, and pathogens to lysosomes. It is a system accountable for perpetually eliminating misfolded proteins or organelles in lysosomes while sustaining differentiation, restructuring, and cellular equilibrium (Dohi et al., 2012). While beneficial in most cases, excessive autophagy may lead to “type 2”or “autophagic” cell death. Most brain ischemia models have confirmed that increased autophagy protects neurons from apoptosis (Yu et al., 2017; Wang et al., 2024). Increasing evidence suggests that autophagy regulates neuronal survival and death, both of which are related to ischemic stroke and I/R injury (Ghavami et al., 2014; Liu K.-Y. et al., 2020). Studies have confirmed that autophagy serves a vital function in safeguarding neurons against apoptosis (Zhang et al., 2019; Sun X. et al., 2020). Many neurons die after CIRI. Nevertheless, in contrast to the ischemic core, neuronal demise in the ischemic penumbra is reversible, rendering it a potential therapeutic target for CIRI (Ferrer and Planas, 2003). The buildup of impaired mitochondria and improperly folded proteins during trauma is a significant factor that leads to neuronal death. The autophagy-lysosome pathway maintains cellular homeostasis, which is crucial for normal cellular functions (Glick et al., 2010). Impairment of this pathway leads to abnormal protein aggregation and mitochondrial impairment, promoting oxidative strain and apoptosis. Earlier research has established that exosomes secreted by bone marrow stem cells enhance neuroprotection through the modulation of autophagy (Zeng et al., 2020). Employing a model of persistent cerebral ischemia, we found that the autophagy-lysosome pathway was impaired 24 h after ischemia (Liu Y. et al., 2017). Research has indicated that lysosomal impairment following ischemic damage interferes with the autophagy-lysosome pathway, resulting in the atypical buildup of autophagosomes (Carloni et al., 2008; Wang et al., 2011; Viscomi et al., 2012). Additionally, research has demonstrated that lysosomal dysfunction is a consequence of ischemic injury, which may manifest as cytoplasmic acidification and/or the rupture or permeabilization of lysosomes (Yamashima, 2000; Yamashima et al., 2003; Li et al., 2014; Zhou et al., 2017). Enhancing the autophagy-lysosome pathway by alleviating lysosomal dysfunction could serve as a potentially effective treatment approach for CIRI. Although autophagy activation may have neuroprotective effects, studies have shown that autophagy inhibitors (such as 3 MA) can prevent programmed necrosis induced by severe global cerebral ischemia (Wang et al., 2011). Therefore, it may be advantageous to regulate autophagy activation and inhibition based on the specific pathological stage.

### 2.4 Cerebral ischemia-reperfusion injury and ferroptosis

Ferroptosis represents a recently discovered mechanism of cellular demise characterized by lipid hydroperoxide accumulation, dependent on iron reaching lethal levels (Dixon et al., 2012; Stockwell et al., 2017). Numerous neurological disorders have been identified, encompassing conditions such as degenerative diseases, traumatic brain injuries, hemorrhagic strokes, and ischemic strokes (Stockwell et al., 2017; Wu et al., 2018). Ischemic stroke induces ferroptosis, evidenced by increased concentrations of lipid peroxidation byproducts are observed alongside reduced levels of antioxidants; however, the application of ferroptosis inhibitors has the potential to ameliorate I/R injury (Alim et al., 2019). Biochemically, brain tissue is abundant in phospholipids, and a notable characteristic of brain injury is lipid peroxidation. Overproduction and harmful buildup of lipid ROS in biological membranes during ischemic stroke lead to glutathione depletion and GPX4 inactivation (Yang and Stockwell, 2016). The overproduction of glutamate by neurons affected by ischemia leads to the inhibition of cystine/glutamate reverse transporters, resulting in glutamate-induced neurotoxicity (Jin et al., 2021). During the pathological advancement of ischemic stroke, the accumulation of iron ions and iron-dependent lipid peroxidation reactions increase (Hanson et al., 2009). Damage to the structure and function of the BBB also promotes the transport of iron ions from the bloodstream into the brain tissue, thereby initiating neuronal ferroptosis (Dixon et al., 2012; Yu and Chang, 2019). Prior research has demonstrated that inhibitors of ferroptosis markedly decrease the volume of infarcts and the extent of neurological impairments, thereby mitigating the effects of CIRI (Ahmad et al., 2014; Tuo et al., 2017). The results of this study reinforce the significant association between ferroptosis and ischemic stroke. Additionally, ferroptosis exhibits a strong connection with various other pathological mechanisms, including inflammatory responses and oxidative stressors (Liu J. et al., 2020; Sun Y. et al., 2020). Thus, dialectically integrating and analyzing ferroptosis with other related pathological processes may aid in identifying effective therapeutic targets for ischemic stroke.

### 2.5 Cerebral ischemia-reperfusion injury and glial cells

**FIGURE 2:**
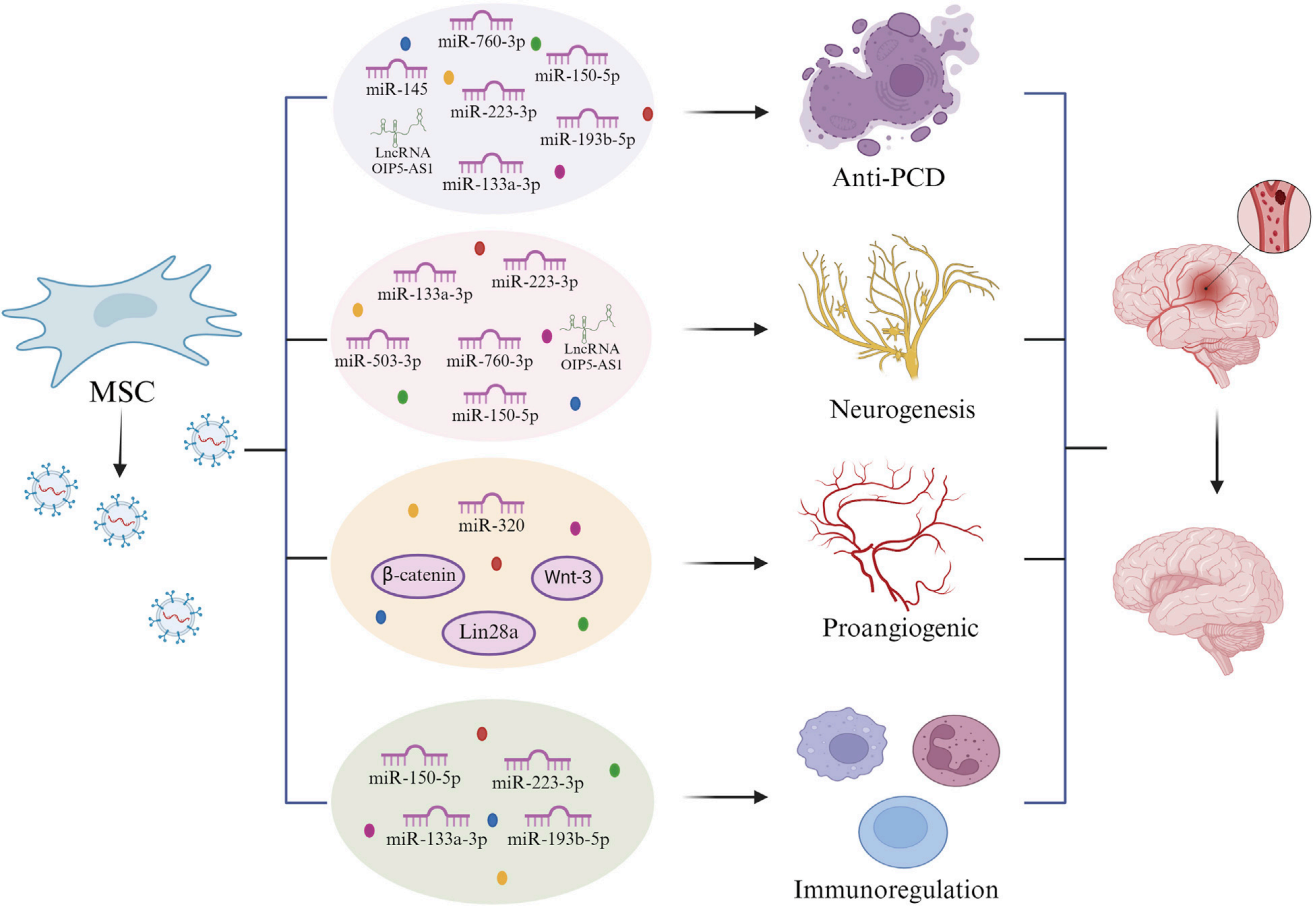
Reparative effect of mesenchymal stem cell-derived exomes on ischemic brain after cerebral ischemia-reperfusion injury. Mesenchymal stem cell-derived exosomes release miRNAs such as LncRNA OIP5-AS1, miR-760-3p, miR-145, miR-150-5p, miR-223-3p, miR-193b-5p, and miR-133a-3p, which exert anti-programmed cell death (anti-PCD) following cerebral ischemia reperfusion injury. Mesenchymal stem cell exosomes exert neuroprotective effects by releasing miR-133a-3p, miR-223-3p, miR-503-3p, miR-760-3p, miR-150-5P, and LncRNA OIP5-AS1. Mesenchymal stem cell exosomes can facilitate the production of brain microvascular endothelial cells via β-catenin, Wnt-3, Lin28a, and miR-320. Mesenchymal stem cell exosomes exert immunomodulatory effects by releasing miR-150-5p, miR-223-3p, miR-133a-3p, and miR-193b-5p.

Microglia are inhabitant immune cells in the central nervous system that regulate brain microenvironment homeostasis and contribute significantly to immune responses (Rivest, 2009; Hu et al., 2015; Xu et al., 2020). Activated microglia rapidly migrate to injury sites and participate in the inflammatory process during CIRI injury. Microglia play a crucial role in stroke, characterized by their dual polarization into pro-inflammatory (M1) and anti-inflammatory (M2) phenotypes (Qin et al., 2019; Leng and Edison, 2021). When CIRI occurs, most microglia are of the M2 type, which helps clear cellular debris. As the condition progresses, microglia polarize to the M1 subtype, exacerbating inflammation in the brain tissue, intensifying the degree of injury, and prolonging the duration of damage (Yenari et al., 2006; Hu et al., 2012; Yang Z. et al., 2015). Increasing evidence suggests that converting M1 microglia to the M2 subtype to suppress inflammation triggered by microglia may be a successful approach to alleviate CIRI (Tikka and Koistinaho, 2001; Jiang et al., 2018; Liu Z. et al., 2017; Wang et al., 2018). Astrocytes serve as supportive matrix cells within the central nervous system, undertaking a multitude of regulatory roles. These include the buffering of extracellular ions, the clearance of amino acid neurotransmitters, the mitigation of excitotoxicity, and the facilitation of synaptic development (Freeman, 2010; Sofroniew, 2020). Studies have confirmed that astrocytes transmit extensive and intricate information with one another and with neurons directly and interactively (Hu et al., 2015). Astrocytes are essential for numerous facets of the development of the nervous system, including synaptic transmission, the modulation of information processing and signaling, maintenance of ion homeostasis, regulation of biochemical pathways, and facilitation of synaptic plasticity. Furthermore, they have the capacity to supply functional mitochondria to neurons, thereby offering protection against ischemic damage. This suggests that the active movement of mitochondria extends beyond intracellular locations, encompassing interactions between different cells as well. The relationship between astrocytes and neurons has been extensively studied (Russo et al., 2021). Consequently, enhancing the performance of astrocytic mitochondria and promoting the transfer of healthy astrocytic mitochondria to neurons could represent effective therapeutic approaches for reducing neuronal injury caused by ischemic stroke (Figure 2).

## 3 Various sources of stem cell-derived exosomes and cerebral ischemia-reperfusion injury

### 3.1 Exosomes from bone marrow mesenchymal stem cells and cerebral ischemia-reperfusion injury

Bone marrow MSCs are non-hematopoietic progenitor cells of the skeletal system that significantly play a role in the hematopoietic microenvironment (Li H. et al., 2016). The process of transplanting of bone marrow MSCs could facilitate the restoration of neurological function following cerebral ischemia (Yu et al., 2021). Recent research has shown that bone marrow MSCs can mitigate CIRI by modulating the PI3K/Akt/mTOR signaling pathway (He et al., 2019). Li et al. demonstrated that exosomes exert anti-apoptotic effects through the Wnt-3a/β-catenin pathway, enhancing the proliferation of microvascular endothelial cells and facilitating angiogenesis may serve as a therapeutic strategy to mitigate the effects of CIRI (Liu et al., 2021). Yang et al. found that exosomes from bone marrow MSCs alleviate CIRI by targeting DAPK2/Akt signaling through miR-133a-3p (Yang et al., 2023). Xie et al. discovered that lncRNA KLF3-AS1, delivered by exosomes from bone marrow MSCs, promotes Sirt1 deubiquitination through the KLF3-AS1/miR-206/USP22 network, reducing inflammation induced by CIRI and providing neuroprotection (Xie et al., 2023). Zhou et al. confirmed that exosomes derived from bone marrow MSCs exhibit a protective effect against CIRI by modulating the expression of FOXO1 via the action of miR-145 (Zhou et al., 2022). Li et al. found that exosomes originating from bone marrow MSCs exhibit a protective effect against CIRI by suppressing TLR5 via the action of miR-150-5p (Li et al., 2022). Additional studies confirmed that exosomes derived from bone marrow MSCs demonstrate a protective effect against cerebral ischemia injury and CIRI through the transfer of miR-193b-5p, thereby inhibiting AIM2 pathway-mediated pyroptosis (Wang et al., 2023a). Exosomes derived from bone marrow MSCs act as crucial mediators of the biological functions of these cells (Li et al., 2020; Zeng et al., 2020), modulating the biological attributes of target cells via intercellular receptor transfer, targeted ligand receptors, RNA molecules, and proteins (Zeng et al., 2020). Prior research suggests that exosomes derived from bone marrow MSCs can alleviate CIRI by modulating autophagy, highlighting their potential role in CIRI.

### 3.2 Exosomes from neural stem cells and cerebral ischemia-reperfusion injury

Neural stem cells can differentiate into neurons and glial cells and be integrated into the synaptic network (Zeng et al., 2021). Owing to their low immunogenicity and widespread availability, stem cells have attracted increasing attention, offering new avenues for future clinical disease treatments (Fu et al., 2017). Recent research has validated that the therapeutic efficacy of neural stem cells is contingent upon paracrine signaling mechanisms, with exosomes serving as vital paracrine mediators (Hwang and Hong, 2017). Exosomes promote communication between cells by delivering proteins, RNA, and microRNAs to neighboring cells (Rong et al., 2019; Zhong et al., 2020). Recent research indicates that exosomes from neural stem cells exhibit neuroprotective effects against CIRI. These effects are primarily manifested as a reduction in infarct area, brain edema, and neuroinflammation, along with an improvement in neurological function. Additionally, exosomes derived from neural stem cells can also regulate the release of inflammatory factors associated with microglia, thereby mitigating inflammation (Zhao et al., 2023). Consequently, exosomes from neural stem cells could exhibit potential therapeutic targets for CIRI.

### 3.3 Exosomes from human umbilical cord blood mesenchymal stem cells and cerebral ischemia-reperfusion injury

Research has confirmed that human umbilical cord blood MSCs are crucial in cell therapy for ischemic stroke (Li et al., 2015). These cells enhance disease progression by either directly differentiating into endothelial cells or via paracrine signaling mechanisms (Xie et al., 2020). Exosomes from human umbilical cord blood MSCs are crucial mediators of paracrine mechanisms, modulating intercellular communication through the transfer of signaling molecules, including circular RNA, long non-coding RNA, and microRNA (Yin et al., 2020). Human umbilical cord blood MSCs exhibit strong secretory functions, releasing therapeutic biomolecules that promote cell repair and growth (Yaghoubi et al., 2019). Recent studies on stroke have demonstrated that stem cell-derived exosomes mediate therapeutic effects (Kim et al., 2020). Feng et al. demonstrated that exosomes originating from mesenchymal stem cells of the human umbilical cord, which contain circDLGAP4, can mitigate cerebrovascular damage by regulating the miR-320/KLF5 signaling pathway, thereby alleviating brain microvascular damage in CIRI (Feng et al., 2023). Therefore, these exosomes can traverse the BBB and mitigate brain damage after ischemic stroke.

### 3.4 Exosomes from adipose-derived mesenchymal stem cells and cerebral ischemia-reperfusion injury

Numerous preclinical and clinical studies have validated the efficacy of MSCs in mitigating neurological impairments post-ischemic stroke (Stonesifer et al., 2017; Chung et al., 2021). A significant portion of this research was focused on adipose-derived MSCs owing to their abundance and accessibility (Bacakova et al., 2018). Numerous researches have shown that adipose-derived MSCs can mitigate ischemic stroke damage through enhancing angiogenesis and synaptic restructuring, diminishing apoptosis, lowering inflammatory elements, and lessening glial scarring (Ishizaka et al., 2013; Jiang et al., 2014; Oh et al., 2015; Chi et al., 2018). Wang et al. demonstrated that miR-760-3p in exosomes prevents ferroptosis by focusing on CHAC1 within neuronal cells. Anti-ferroptosis strategies are considered effective measures to improve CIRI (Wang et al., 2023b). However, the survival of adipose-derived MSCs post-transplantation is significantly challenging. Research has verified that these cells mainly achieve their healing impacts via paracrine routes, with exosomes from adipose-derived MSCs being considered as potential alternatives (Oh et al., 2015). The therapeutic benefits of exosomes derived from adipose-derived MSCs have been investigated and confirmed in hemorrhagic stroke, and their inhibitory effects on ferroptosis have also been identified (Yi and Tang, 2021; Lin et al., 2022). Therefore, we hypothesize that adipose-derived MSCs may exert their therapeutic impacts on CIRI through ferroptosis inhibition.

### 3.5 Plasma exosomes and cerebral ischemia-reperfusion injury

**FIGURE 3:**
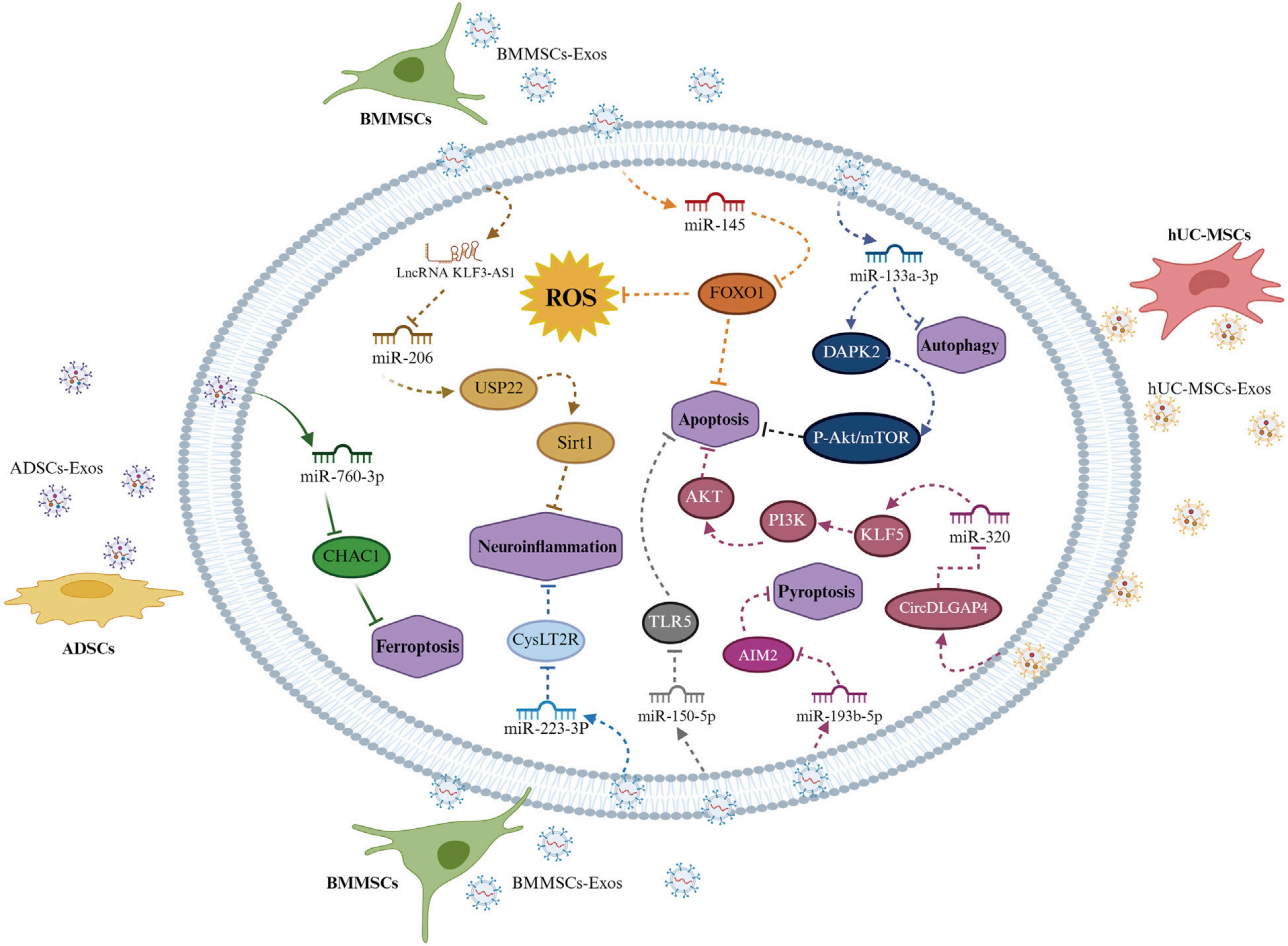
Exosomes from different mesenchymal stem cells mediate apoptosis in brain cells after cerebral ischemia-reperfusion injury. Bone marrow mesenchymal stem cell exosomes inhibit neuronal apoptosis by targeting the DAPK2/Akt signaling pathway through miR-133a-3p. They also promote Sirt1 deubiquitination, which alleviates inflammation damage induced by cerebral ischemia-reperfusion injury through the lncRNA KLF3-AS1/KLF3-AS1/miR-206/USP22 pathway. They downregulate FOXO1 through miR-145 to inhibit neuronal apoptosis and reduce neuroinflammatory damage via the miR-223-3P/CysLT2R pathway. Mesenchymal stem cells also inhibit neuronal apoptosis through the miR-150-5p/TLR5 signaling pathway and suppress neuronal pyroptosis via the miR-193b-5p/AIM2 pathway. Exosomes that originate from mesenchymal stem cells in human umbilical cord blood mitigate cerebrovascular damage through the modulation of the miR-320/KLF5 signaling pathway. Adipose-derived mesenchymal stem cell exosomes exert neuroprotective effects by inhibiting ferroptosis through the miR-760-3p/CHAC1 pathway.

Among these various sources of exosomes, plasma contains exosomes from all cell sources, which are believed to play pathological roles in various diseases (Bei et al., 2017; Kang et al., 2019). Compared to the small number of exosomes secreted by cells, a large quantity of plasma exosomes can be easily obtained through the release of reticulocytes. Additionally, plasma exosomes’ safety is greatly improved by their absence of immunostimulatory and cancer-inducing properties (Blanc et al., 2005). Research has verified the presence of HSP70 in human plasma exosomes, which is exported to the extracellular space and plays a role in regulating ROS(Calabrese et al., 2004; Cheng et al., 2014). A multitude of research has shown that plasma exosomes can ameliorate cerebral ischemia-reperfusion injury by markedly decreasing the size of brain infarcts, enhancing neurological performance, diminishing apoptosis, and mitigating oxidative stress (Luo et al., 2019). Plasma exosomes have been thoroughly studied as potential therapeutic agents and biomarkers for ischemic stroke (Zhou et al., 2018; Otero-Ortega et al., 2019). These include a range of operational proteins produced by cells that donate. Recent research indicates that circulating exosomal proteins might influence target receptor cells’ functionality and contribute to neuroprotection in I/R injury. However, the makeup of these proteins is still not well understood, and the specific signaling routes they stimulate are yet to be thoroughly investigated (Shi et al., 2019) (Figure 3).

## 4 Summary and outlook

The development of CIRI involves various complex pathophysiological processes. Stem cell transplantation for ischemic stroke treatment can enhance endogenous neural regeneration and brain tissue self-repair. However, therapeutic efficacy is directly affected by the heterogeneity of stem cells. Exosome-based acellular therapies have gained attention owing to their ability to mitigate risks associated with direct stem cell therapies, such as low survival rates, strong immune rejection, and high mutagenic tumorigenicity. The role of exosomes in transporting functional proteins, lipids, and nucleic acids has garnered increasing attention. Numerous studies have confirmed that exosomes alleviate CIRI through various mechanisms, suggesting broad application prospects. The molecular mechanisms by which extracellular vesicles regulate CIRI include: inhibition of programmed cell apoptosis (by proteins such as HSP70 and Wnt-3) to promote angiogenesis and neural repair, regulation of M1/M2 polarization of microglia to alleviate inflammation, and maintenance of blood-brain barrier integrity through circRNAs (such as circDLGAP4). However, the precise mechanisms through which exosomes affect CIRI are not yet fully understood. Therefore, further in-depth studies are needed to clarify this relationship. Standardized preparation of extracellular vesicles, targeted delivery strategies, and clinical translation pathways should also be explored.
